# Transcriptomic profiling of programmed cell death 1 (PD-1) expressing T cells in early rheumatoid arthritis identifies a decreased CD4 + PD-1 + signature post-treatment

**DOI:** 10.1038/s41598-023-29971-5

**Published:** 2023-02-17

**Authors:** Katie Lowe, Annabelle Small, Qingxuan Song, Ling-Yang Hao, William Murray-Brown, Susanna Proudman, Malcolm D. Smith, Sunil Nagpal, Mihir D. Wechalekar

**Affiliations:** 1 Rheumatology Synovial Tissue Translational Research Group, Flinders University, Adelaide, South Australia 5042 Australia; 2 Discovery Immunology Janssen R&D, 1400 McKean Road, Spring House, PA 19477 USA; 3 Department of Rheumatology, Royal Adelaide Hospital, Adelaide, Australia; 4 Discipline of Medicine, University of Adelaide, Adelaide, Australia; 5 Department of Rheumatology, Flinders Medical Centre, Adelaide, South Australia 5042 Australia

**Keywords:** Immunology, Rheumatology

## Abstract

Programmed cell death protein 1 (PD-1)-expressing T cells are expanded in individuals with established rheumatoid arthritis (RA). However, little is known about their functional role in the pathogenesis of early RA. To address this, we investigated the transcriptomic profiles of circulating CD4^+^ and CD8^+^ PD-1^+^ lymphocytes from patients with early RA (n = 5) using fluorescence activated cell sorting in conjunction with total RNA sequencing. Additionally, we assessed for alterations in CD4^+^PD-1^+^ gene signatures in previously published synovial tissue (ST) biopsy data (n = 19) (GSE89408, GSE97165) before and after six-months of triple disease modifying anti-rheumatic drug (tDMARD) treatment. Comparisons of gene signatures between CD4^+^PD-1^+^ vs. PD-1^−^ cells identified significant upregulation of genes including *CXCL13* and *MAF*, and in pathways including Th1 and Th2, cross talk between dendritic cells and NK cells, B cell development and antigen presentation. Gene signatures from early RA ST before and after six-month tDMARD treatment revealed downregulation of the CD4^+^PD-1^+^ signatures following treatment, identifying a mechanism through which tDMARDs exert their effect by influencing T cell populations. Furthermore, we identify factors associated with B cell help that are enhanced in the ST compared with PBMCs, highlighting their importance in driving synovial inflammation.

## Introduction

Rheumatoid arthritis (RA) is a chronic, inflammatory autoimmune disease which leads to joint damage, disability, and a reduction in quality of life. It is characterised by synovial membrane inflammation, with inflammatory immune cell infiltration into the synovial tissue (ST), and strong evidence suggests that the immune cell types that invade the ST heavily influence disease prognosis and treatment success^1^. In particular, CD4^+^ and CD8^+^ T lymphocytes have been implicated in RA pathogenesis and dominate the lymphocytic infiltrate of RA^2,3^. However, the precise populations of these cells responsible for driving early disease and their origins remain somewhat mysterious and thus, further definition into these subsets is needed before we can fully understand their role in RA.

Programmed cell death protein 1 (PD-1) is an immune checkpoint receptor which is expressed predominantly by T lymphocytes^4^. Interaction with either of its ligands, programmed death ligand 1 or 2 (PD-L1 or PD-L2, respectively) elicits an inhibitory response in the PD-1-expressing T cells which in turn induces the inactivation of T cell receptor and costimulatory signalling cascades^5^. This can result in T cell apoptosis, anergy, or exhaustion^6^. In cancer patients treated with checkpoint inhibitors that block the PD-1 pathway, inflammatory arthritis as an adverse event has been reported to occur in ~ 4% of patients. Additionally, PD-1-expressing T cells are abundant in both the peripheral blood and in the synovium of patients with established, seropositive RA, consistent with disorders involving long-term antigen exposure^7–11^, further indicating a role of the PD-1 pathway in the development of RA^12^. Yet these PD-1^+^ subsets are not exhausted; CD4^+^CXCR5^+^PD-1^+^ T follicular helper (Tfh) and CD4^+^CXCR5^−^PD-1^+^ T peripheral helper (Tph) cells have been proposed to contribute to the pathogenesis of RA by secreting B cell-promoting factors and promoting B cell differentiation and maturation, thus driving autoantibody production^11,13^.

While research into the role of CD4^+^ T cells in autoimmune conditions and in inflammatory arthritis is abundant, their regulators and specific role in driving early RA remain to be elucidated. Meanwhile, despite the presence of CD8^+^ PD-1^+^ cell populations in peripheral blood from established RA patients, albeit at a lower frequency than in synovial fluid (SF)^10^ and a high frequency of CD8^+^ PD-1^+^ cells in early RA ST^14^, similar studies into the role of CD8^+^ T cells in RA, also remain scarce^14^. In order to address these gaps, we isolated CD4^+^/CD8^+^ PD-1^+^/^−^ cell populations from the peripheral blood of early RA patients by fluorescence activated cell sorting (FACS) and investigated their genetic profiles by RNA sequencing (RNAseq). Furthermore, we utilised existing total RNAseq data from a clinically well-defined, seropositive, uniformly-treated, cohort of 19 early RA patient biopsy samples, pre- and post- 6-months of triple disease modifying anti rheumatic drug (tDMARD) treatment^15^, and assessed for gene expression profiles of PD-1^+^ cells within the ST. Our results identified substantial differences between CD4^+^ PD-1^+^ populations in the circulation compared with PD-1^−^ cells and identified significant enrichment of genes involved in providing B cell help. Finally, we observed a decrease in CD4^+^ PD-1^+^ cell gene signatures with treatment, suggesting a mechanism by which tDMARDs exert their effects. Together, our findings highlight the importance of PD-1-expressing T cells in early RA and identify the potential use of these gene expression profiles as markers of response to treatment.

## Results

### Transcriptomic analysis reveals significant differences in gene signatures between circulating PD-1^+^ vs PD-1^−^ lymphocytes

**Figure 1:**
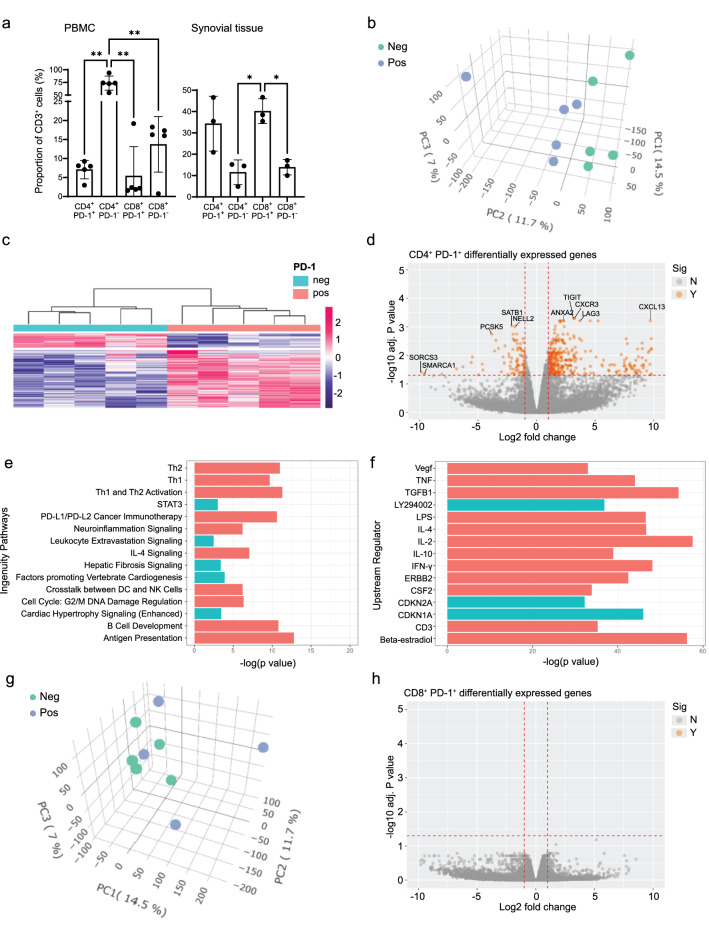
Gene expression profiling separates CD4^+^ but not CD8^+^ PD-1^+^ cells from PD-1^−^ cells in early RA patients. (**a**) T cell proportions obtained from peripheral blood (n = 5) and synovial tissue (n = 3) by FACS. Data is expressed as proportion of CD3^+^ T cells ± s.d. Comparisons were made by one-way ANOVA followed by Tukey’s Multiple Comparison Test, *p < 0.05, **p < 0.01. (**b**) PCA plot of CD4^+^ cells sorted from early RA PBMCs (n = 5). Samples are coloured by PD-1 positivity, (PD-1^+^ cells are shown in blue, (pos), and PD-1^−^ cells are shown in green (neg). (**c**) Heatmap of significantly differentially expressed genes and hierarchical clustering analysis of CD4^+^ cells sorted from early RA PBMCs (n = 5). Each column represents one patient sample (**d**) Volcano plot depicting the log (fold-changes) and p-values for comparison of CD4^+^PD-1^+^ versus CD4^+^PD-1^−^ cells sorted from early RA PBMCs (n = 5); significantly modulated genes are represented in orange (Sig: Y); grey represents not significant (Sig: N), with fold change > 2 (or < 0.5) and FDR < 0.05 as cut-off. (**e**) Top 15 Ingenuity Pathways expressed in CD4^+^PD-1^+^ versus CD4^+^PD-1^−^ cells sorted from early RA PBMCs (n = 5). Red indicates upregulated pathways. Blue indicates down regulated. (**f**) Top 15 Upstream Regulators of CD4^+^PD-1^+^ cells sorted from early RA PBMCs (n = 5), as predicted using Qiagen Ingenuity Pathway Analysis (IPA). Red indicates positive regulators, blue indicates negative regulators. (**g**) PCA plot of CD8^+^ cells sorted from early RA PBMCs (n = 4–5). Samples are coloured by PD-1 positivity, (PD-1^+^ cells are shown in blue, (pos), and PD-1^−^ cells are shown in green (neg). (**h**) Volcano plot depicting the log (fold-changes) and p-values for comparison of CD8^+^PD-1^+^ versus CD8^+^PD-1^−^ cells sorted from early RA PBMCs (n = 4–5); significantly modulated genes are represented in orange (Sig: Y); grey represents not significant (Sig: N), with fold change > 2 (or < 0.5) and FDR < 0.05 as cut-off.

Peripheral blood mononuclear cells (PBMCs) were obtained from 5 seropositive early RA patients prior to tDMARD treatment commencement. Patient characteristics are shown in Supplementary Table 1, column 2. Lymphocytes were sorted into CD4^+^/CD8^+^ PD-1^+^ and PD-1^−^ populations (Supplementary Fig. S1), and total RNAseq analysis was performed. CD4^+^PD-1^−^ cells were the dominant population of T cells from the peripheral blood of early RA patients (Fig. 1a). Upon comparison, CD4^+^PD-1^+^ and CD4^+^PD-1^−^ cells clustered separately by principal component analysis (PCA) (Fig. 1b) and hierarchical clustering (Fig. 1c). Further comparison between CD4^+^PD-1^+^ and CD4^+^PD-1^−^ cells revealed 347 significantly enriched genes, including factors associated with B cell help; cytokines such as *CXCL13* and *IFNG;* inhibitory signalling molecules including *CTLA-4, LAG3* and *TIGIT*, and several non-coding miRNAs including *MIR155HG* (Fig. 1d, Table 1, Supplementary Data). A further 85 genes were downregulated, including *IL-4R, CCR7, and SATB1*. Ingenuity pathway analysis (Qiagen) revealed significantly modulated pathways (Fig. 1e), including those involved in the Th2 pathway, Th1 pathway, cross talk between dendritic cells and NK cells, B cell development and Antigen presentation, (Fig. 1e, Supplementary Data). Additionally, further analysis predicted upstream regulators of the observed gene signatures, including IL-4, IL-10 IFN-γ, TNF and TGF-β1 (Fig. 1f). In contrast, PCA of CD8^+^PD-1^+^ and CD8^+^PD-1^−^ cells did not reveal separate clusters (Fig. 1g) and differential gene expression analysis of CD8^+^ cells demonstrated no significantly differentially expressed genes when comparing CD8^+^PD-1^+^ versus CD8^+^PD-1^−^ cells (Fig. 1h), suggesting that peripheral blood CD8^+^ PD-1^+^ cells are not a true cell subtype present in the periphery of early RA patients.

**Table 1:** Table summarising significantly enriched genes of interest in CD4^+^ PD-1^+^ isolated from baseline RA PBMCs, as compared with CD4^+^ PD-1^−^ cells.

CD4^+^ PD-1^+^		
Gene	Fold change	Adjusted P-value
IL21-AS1	1378.758	0.000180919
CXCL13	844.990	1.29E−07
TNFAIP6	286.642	0.00030192
IFNG	16.942	0.000357649
MIR4435-2HG	14.149	4.40E−06
TOX	13.736	7.93E−05
LAG3	12.821	9.52E−08
GZMK	12.601	2.47E−06
MIR155HG	9.819	0.000115393
TIGIT	9.491	1.89E−08
CXCR3	9.019	3.30E−08
TNFRSF18	7.221	2.86E−06
GZMA	6.258	0.000224941
TBXAS1	5.841	9.94E−05
IRF5	5.713	9.84E−05
ANXA2	5.005	6.84E−08
MAF	4.520	2.87E−05
TNFRSF4	4.326	3.22E−05
SLAMF7	4.301	0.000774025
ANXA5	3.298	3.21E−05
ANXA4	3.226	0.000112796
CTLA-4	3.159	4.44E−06
SLAMF1	2.409	3.42E−05
SLAMF6	2.310	0.000128985
CD84/SLAMF5	2.750	0.000468619
SH2D2A	2.244	5.20E−05
LINC01550	0.476	0.000104674
IL4R	0.465	6.23E−06
CCR7	0.313	3.77E−06
SATB1	0.228	3.95E−07

FACS of disaggregated cells from the early RA ST revealed that PD-1^+^ cells were the dominant T cell population within ST (Fig. 1a). CD8^+^PD-1^+^ were significantly enriched when compared to CD8^+^PD-1^−^ cells and there was no significant difference in the numbers of CD4^+^PD-1^+^ and CD8^+^PD-1^+^ cells within the ST. Unfortunately, due to the limited cell numbers isolated from paired ST, RNAseq of these samples could not be completed.

### Gene expression profiles of CD4^+^PD-1^+^ are enriched in early RA and decrease following tDMARD treatment

**Figure 2:**
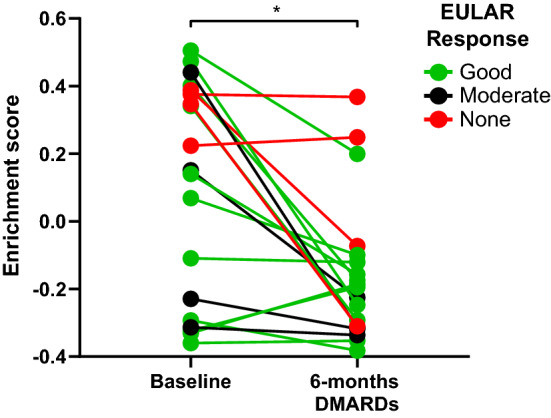
Effect of 6-month tDMARD treatment on the GSVA enrichment scores of gene signatures of CD4^+^ PD-1^+^ cells from ACPA + RA ST (n = 19, GSE89408, GSE97165). Grey lines indicate the pairwise samples from sample donors before and after treatment.

In order to further investigate the involvement of CD4^+^ PD-1^+^ cells in early RA, we sought to examine their gene signatures in ST and assess the correlation of their gene signatures in response to treatment. To this end, we utilised all 347 significantly upregulated genes from the total RNA sequencing data (summarised in Supplementary Data) to create a gene signature and enriched for this signature in the ST of 19 early RA patients prior to, and following 6 months of triple DMARD therapy (GSE89408, GSE97165)^15^. Patient characteristics are shown in Supplementary Table 1, columns 3 and 4.Within our cohort (n = 19), there were 11 good responders, 6 moderate responders, and 2 non-responders to treatment. We observed baseline enrichment consistent with infiltration of CD4^+^PD-1^+^ cells into the tissue. Following 6 months of treatment with triple DMARDs we observed a significant reduction in the gene signatures of CD4^+^PD-1^+^ cells (Fig. 2) (P value = 0.03). Due to a low cohort sample size, we were unable to make a statistical comparison of the CD4^+^PD-1^+^ gene expression enrichment scores with clinical outcome following 6-month triple DMARD treatment, however there was no clear association between the reduced CD4^+^PD-1^+^ signature and treatment response as both responders and non-responders to tDMARD therapy exhibited a decrease in the CD4^+^PD-1^+^ gene signature.

### *IL21*,* c-MAF*, and *CXCL13* are upregulated in the RA ST relative to PBMCs

**Figure 3:**
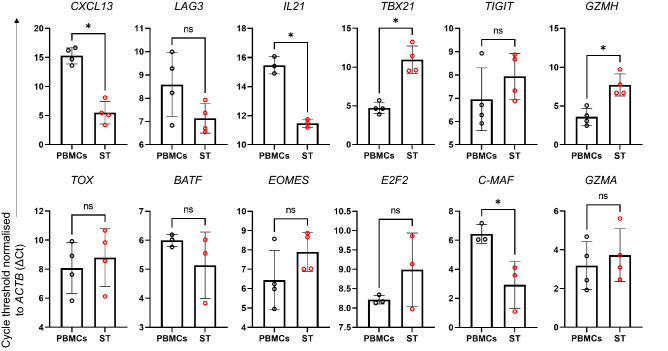
Quantitative RT-PCR analysis of specific genes from treatment-naïve early RA ST biopsy samples versus matched PBMCs from the same individuals (n = 3–4). *ACTB* was used as housekeeping control gene. Data is expressed as threshold cycle normalised to housekeeping gene (∆Ct), as mean ± s.d. Comparisons were made by two-tailed Mann–Whitney U test, ns-not significant; *p < 0.05.

To further examine the cellular interactions occurring within early RA patients and to support the findings from our RNA sequencing, we analysed the expression of a panel of 12 genes in matched baseline RA PBMC and ST samples from the same individuals whose samples were used for the analysis presented in Fig. 1. We observed significantly lower ∆Ct values of *IL21, MAF,* and *CXCL13* in ST compared with PBMCs, which reflect higher expression levels in the ST (Fig. 3). There was no significant difference in the expression of *LAG3* and *TIGIT* between PBMCs and ST. Higher ∆Ct values of *TBX21* and *GZMH* were observed, indicating decreased relative expression in the ST, however, this may be due to the fact that total RNA, rather than RNA from sorted CD4^+^PD-1^+^ populations was assessed, and thus, may not represent a true reflection of expression levels by CD4^+^PD-1^+^ cells.

## Discussion

CD4^+^ PD-1^+^ and CD8^+^ PD-1^+^ cells have been detected in peripheral blood and ST in patients with RA, and accumulation of these populations can occur in ST, with disease progression^8,16^. Profiling cells from the peripheral blood of early RA patients provides us with a picture of the circulating cell populations in chronic disease and could provide us with targets for treatment before disease progression. Our results demonstrate significant differences in the gene expression profiles between circulating PD-1^+^ and PD-1^−^ CD4 populations in early RA patients before the initiation of treatment. The decreased CD4^+^ PD-1^+^ gene signature post-treatment suggests that modulation of these cells is one of the mechanisms by which triple DMARDs may exert their effects. The unique gene expression profiles exhibited by CD4^+^ PD-1^+^ cells highlight multiple pathways by which these cells may contribute to disease pathogenesis, including the ST damage seen in RA patients.

When comparing PD-1^+^ and PD-1^−^ cells, we found that CD4^+^PD-1^+^ cells from the peripheral blood of patients with early RA exhibit enhanced expression of *CXCL13* and *MAF*, factors associated with B cell help (Table 1 and Supplementary Data). This is supported by our detection of *CXCL13*, *MAF* and *IL-21* by PCR, in PBMCs, prior to treatment. The upregulation of these factors in circulating CD4^+^PD-1^+^ cells and within the inflamed ST relative to PBMCs (Fig. 3), indicates that these factors may be involved in both early and established RA (as previously described) and that they may play different roles in different anatomical locations, for example recruitment of B cells in ST. *MAF* is a transcription factor which is expressed by both Tfh and Tph cells^17^, where it regulates the expression of cytokines which promote B cell help, including IL-4, IL-21 and IL-10^17,18^. It is likely that the expression of these factors is reflective of the circulating and tissue-resident Tfh and Tph populations, non-exhausted memory cells, which have previously been demonstrated to be expanded in established RA and function through their production of high levels of IL-21 and CXCL13^11,19^. This is further supported by the increased expression of *KLRB1*/CD161 and *CXCR3,* and decreased expression of *CCR7* which characterises memory cells capable of producing IFNγ, IL-4 and IL-5^20^^21^.

In addition to these factors, several other genes which have also been implicated in T and B cell interactions, including multiple from the signalling lymphocytic activation molecules (SLAM) family were upregulated in CD4^+^PD-1^+^ cells (Table 1). Increased SLAMF1/CD150 expression on T cells has been described in patients with Systemic Lupus Erythematosus (SLE)^22^ and its engagement diminishes the production of IL-6 and plasmablast differentiation^23^. Increased expression of *SLAMF5* and *SLAMF6* is consistent with the elevated expression detected in CD4^+^PD-1^hi^ cells from established RA patients where plasma cell differentiation and IgG production were abrogated upon SLAMF6 antibody blockade^11^. Together, these findings support a role of CD4^+^PD-1^+^ Tfh and Tph cells in driving early RA and indicate multiple routes of potential B cell interaction.

Additional upregulated genes encoding cell surface receptors on CD4^+^PD-1^+^ cells included those from the annexin family. Annexin A2, encoded by *ANXA2*, binds the discoidin domain receptor 2 on fibroblast-like synoviocytes, and has been detected in RA ST where it has been implicated in arthritic joint destruction^29^. Its expression on peripheral T cells here may provide a link between circulating inflammatory T cells and localised joint destruction. CD74, which binds macrophage migration inhibitory factor (MIF) also exhibited increased expression on CD4^+^PD-1^+^ cells in relation to CD4^+^PD-1^−^ cells. MIF/CD74 signalling is extremely varied and can promote protective or inflammatory pathways involving enhanced expression of IL-6 or TNF. CD74 is involved in proliferation, survival and wound repair^30^ and increased expression has been demonstrated on B cells in a mouse model of SLE^31^ and in patients with inflammatory bowel disease^32^. Signalling via CD74 on T cells in the context of RA, warrants further investigation to determine if this receptor is contributing to the systemic inflammation seen in RA. The increased expression of cell surface molecules involved in cellular interactions with other cell types provides multiple pathways which could be used as targets for future treatments.

Negatively associated regulators of CD4^+^PD-1^+^ cells detected here include *SATB1* and several non-coding RNAs. This was not surprising due to the previous description of increased PD-1 with decreasing *SATB1*^24^ and corresponds with our increased detection of *mir155HG*, which can regulate *SATB1* expression^25^. Long non-coding RNAs or microRNAs can inhibit transcription or translation of genes, and the increased expression of mir155 detected here is consistent with the upregulation of mir155 previously detected in PBMCs from established RA patients^26^. Our data indicates mir155 is present in CD4^+^PD-1^+^ cells in early RA patients and may be involved in a feedback loop upregulating *ANXA2*, which has previously been described in glioblastoma^27^.

Multiple soluble factors have been detected in the serum of RA patients but the origin of these and their involvement in disease pathogenesis is often unclear. Elevated levels of connective tissue growth factor (*CTGF*) have been described in serum from RA patients and detected in RA ST^28^ and the increased *CTGF* detected here suggests that this may originate from CD4^+^PD-1^+^ cells. CTGF can upregulate TGF-β1, which contributes to fibrosis in Graves’ Ophthalmopathy^29^. As TGF-β1 is an important cytokine for the differentiation of Tfh cells and is also able to induce the differentiation of CXCL13-producing Tph-like cells^30,31^, it was not surprising that this was a predicted upstream regulator of CD4^+^PD-1^+^ cells.

Interestingly, several genes described here in circulating CD4^+^PD-1^+^ cells from early RA patients prior to treatment have been described in established RA patients, including those from the SLAM, Annexin and DUSP families. Of the 66 genes differentially expressed in CD4^+^PD-1^hi^ cells from patients with established RA^11^, 22 were also found to be differentially expressed in our data from early RA patients. Consistent expression throughout disease progression highlights the importance of these genes in disease pathogenesis. Earlier targeting of these genes and their signalling pathways may thus provide more effective treatments.

Our data show significant enrichment of *TOX*, *LAG3*, *CTLA4*, and *TIGIT* (Table 1) in CD4^+^PD-1^+^ cells, markers typically associated with T cell exhaustion^32^. However, while we observed enrichment of previously published^33^ exhaustion signatures (Supplementary Fig. S2), these ‘traditional’ markers of exhaustion are also expressed by effector cells in states of prolonged antigen exposure^34^. The expression of similar levels of *LAG3* and *TIGIT* by PD-1^+^ PBMCs and on ST cells indicates that certain stimulatory signals begin within the periphery and continue to be imposed on these cells within the localised inflammatory environment. Continuous exposure to antigens in inflammatory environments can result in dampening of particular cellular functions such as proliferation or cytokine release, however the enhanced expression of *IL21* and *CXCL13* indicates that not all functions within PD-1^+^ cells have been compromised and suggests that in the context of early RA examined here, these cells are contributing to disease pathogenesis.

In contrast to the initial characterisation of CD4^+^ PD-1^+^ Tph cells in established RA^11^, our results demonstrate the expression of both *CTLA4* and *LAG3* by PD-1^+^ cells, and this finding is supported by recently published single-cell analyses^35^. Differences in the findings may be due to our isolation of CD4^+^ PD-1^+^ cells, which are not specifically Tph PD-1^hi^CXCR5^−^ CD4^+^ cells, or due to differences in signalling with disease progression and cellular localisation. The expression of multiple inhibitory molecules in the periphery of early RA patients demonstrates the complexity of RA and the diversity of signalling pathways that may be involved in regulating this disease. This lack of differences in gene expression when comparing CD8^+^ PD-1^+^ and CD8^+^ PD-1- cells within the peripheral blood further highlights that diversity in signalling molecules that may be seen in early RA, however it indicates that not all cell markers and cell subsets that can be detected in early RA are true subpopulations which may be involved in disease pathogenesis. Little is known about the role of CD8^+^PD-1^+^ cells in RA and while these cells have been reported to be decreased in the circulation of patients with RA compared with healthy controls^36^, in the ST, our results and those from others show that they are abundant^10,14^. Additionally, non-exhausted CD8^+^PD-1^+^ T cells are also expanded within the synovial fluid of juvenile idiopathic arthritis patients^37^. The accumulation of CD8^+^ PD-1^+^ in ST indicates that different inflammatory mechanisms are in place compared to the peripheral blood here, but further analysis of these ST cells is required to determine their exact role.

Upon analysis of our previously published bulk RNA sequencing of ST results, we determined that prior to treatment, the gene signatures of CD4^+^PD-1^+^ were enriched, consistent with baseline infiltration of these cells into the inflamed ST. The finding that these signatures decrease following treatment suggests the pathogenic nature of these cells, and potentially identifies a mechanism through which tDMARD therapy exerts its therapeutic action. Indeed, CD4^+^ PD-1^+^ T cell subsets (Tfh and Tph cells) have been implicated as major drivers of RA pathogenesis and have been identified as adept promotors of B cell responses^11,19^. To further clarify the role of CD4^+^ PD-1^+^ T cell subsets in relation to treatment response, the analysis of a larger patient cohort is required.

The performed study is not without limitations, one of which is that this work was performed with a limited sample size. Future work examining interactions between CD4^+^ PD-1^+^ T cells and other cell types will require assays to be performed using a larger number of samples. Despite the dominance of CD4^+^ and CD8^+^ PD-1^+^ cells within ST, due to low cell yield from disaggregated biopsy samples, we were unfortunately unable sort and isolate these cells to examine any significantly modulated genes in sorted CD4^+^/CD8^+^ PD-1^+^/PD-1^−^ populations. This may be due to lower levels of inflammation and cellular infiltration in early RA, compared to patients with established disease. Future studies will need to harness advances in technology which allow the examination of individual cells and specific cell populations such as PD-1^+^ cells in situ.

Furthermore, to conduct our qRT-PCR analysis, we assessed total RNA from isolated PBMCs and from matched ST biopsy samples. Due to expected low cell yields, we elected not to sort these samples into CD4^+^/CD8^+^ PD-1^+^/PD-1^−^ populations. However, despite this, we observed increased levels of *CXCL13*, *IL21*, and *MAF* in the ST relative to matched PBMC samples, which correlated with our RNAseq comparisons of PD-1^+^ versus PD-1^−^ T lymphocytes.

Together, our findings highlight the importance of PD-1-expressing T cells in early RA and identify the potential use of these gene expression profiles as markers of response to treatment. Further examination into the expression of these molecule and their corresponding ligands at the protein level could provide insights into pathway dysregulation and provide other targets and opportunities for disease control in patients for whom tDMARD therapy is ineffective.

## Methods

### Human subjects

Arthroscopic synovial biopsies and venous blood were collected from a relatively homogeneous cohort (all patients were seropositive for rheumatoid factor (RF) and anti-citrullinated peptide antibody (ACPA)) of treatment-naïve patients with early RA (defined as within 12 months of onset of symptoms and fulfilling the 2010 ACR/EULAR). For assessment of CD4^+^ PD-1^+^ T cell signatures in ST, we utilised a previously published RNAseq dataset obtained from the ST of early RA, ACPA + patients pre- and post- treatment. All patients received a protocolised treatment regimen comprised of tDMARDs (Methotrexate, Sulfasalazine, and Hydroxychloroquine); treatment protocol included a pre-specified escalation strategy aiming for remission. Demographic and patient characteristics for the PBMC and ST pre- and post- tDMARD treatment cohorts are shown in Supplementary Table S1.

### Purification of peripheral blood mononuclear cells

Peripheral blood mononuclear cells (PBMC) were isolated from patient whole blood samples using Lymphoprep™ (StemCell Technologies, Vancouver, Canada). Briefly, blood was diluted 1:1 in phosphate buffered saline (PBS) prior to being layered onto Lymphoprep™ and centrifuged for 20 min at 500×*g* with no brake. Following centrifugation, the PBMC-containing band was aspirated and washed in PBS (300×*g*, 10 min). Cells were then counted using a haemocytometer and viability judged by their ability to exclude trypan blue. For cryopreservation, cells were cryopreserved in freezing media containing 10% foetal calf serum (FCS), 80% RPMI-1640 media, and 10% DMSO. Cells were incubated in a ‘Mr. Frosty’ Freezing Container (ThermoFisher Scientific, Waltham, MA) overnight in a − 80 °C freezer, before being transferred into liquid nitrogen for long-term storage. Prior to use, cryopreserved PBMCs were removed from storage and thawed rapidly at 37 °C before washing in RPMI-1640. Cells were counted and viability assessed by the trypan blue-exclusion method prior to application.

### Flow cytometry and cell sorting

Synovial cell suspensions and matched fresh PBMC samples were stained with Zombie UV® (BioLegend, San Diego, CA) for 15 min in serum-free PBS prior to washing in 2 mL PBS (500×*g*, 5 min). Cell surface was then stained for 30 min with an antibody cocktail consisting of; anti-CD45RO-BUV395 (UCHL1), PD1-BV421 (EH12.1), CD3-PerCP-Cy5.5 (SK7), ICOS-PE (DX29), CD8-Alexa Fluor® 647 (RPA-T8), CD4-Alexa Fluor® 700 (SK3), CD20-APC-H7 (L27), TIGIT-PE-Cy7 (A15153G), CD38-BB515 (HIT2) (all from BD Biosciences, Franklin Lakes, NJ), and CXCR5-PE/Dazzle™ 594 (J252D4; BioLegend, San Diego, CA) in Brilliant Stain Buffer (BD Biosciences, Franklin Lakes, NJ). Following washing in PBS + 2% FCS, cells were resuspended in PBS + 2% FCS ready for FACS acquisition and sorting on a BD FACSAria™ Fusion flow cytometer. Populations of interest were collected in 1.5 mL Eppendorf tubes prior to storage.

### Total RNA sequencing

#### mRNA purification, NGS library preparation

Total RNA was extracted and processed for library construction by Cofactor Genomics (St. Louis, MO) according to the following procedure: briefly, total RNA was extracted from cell pellets using the protocol for Arcturus PicoPure™ RNA Isolation Kit (Thermo Fisher, Waltham, MA). Total RNA was reverse-transcribed using an Oligo(dT) primer, and limited cDNA amplification was performed using the SMARTer® Ultra® Low Input RNA Kit for Sequencing—v4 (Takara Bio USA, Inc., Mountain View, CA). The resulting full-length cDNA was fragmented and tagged, followed by limited PCR enrichment to generate the final cDNA sequencing library (Nextera® XT DNA Library Prep, Illumina, San Diego, CA). Libraries were sequenced as single-end 75 base reads on a NextSeq500 (Illumina, San Diego, CA) following the manufacturer’s protocols.

#### Sequence data quality control

Raw sequence data in FASTQ format were assessed for quality (FastQC, http://www.bioinformatics.babraham.ac.uk/projects/fastqc/) and ribosomal RNA content (sortmeRNA, http://bioinfo.lifl.fr/RNA/sortmerna/).

### Total RNA isolation, cDNA generation, and quantitive real-time polymerase chain reaction

Briefly, total RNA was extracted from either stored ST biopsy samples or PBMCs using the AllPrep DNA/RNA Mini Kit (Qiagen, Hilden, Germany) according to the manufacturer’s instructions. cDNA was prepared using 200 ng RNA using the iScript™ cDNA Synthesis Kit (Bio-Rad, Hercules, CA). Quantitive reverse transcription PCR analysis was performed using TaqMan™ Gene Expression Master Mix (ThermoFisher Scientific, Waltham, MA) and the following TaqMan™ Gene Expression Assays; c-MAF (Hs00193519_m1), GZMA (Hs00989184_m1), GZMH (Hs00277212_m1), BATF (Hs00232390_m1), EOMES (Hs00172872_m1), TIGIT (Hs00545087_m1), TOX (Hs04264584_m1), E2F2 (Hs00918090_m1), IL21 (Hs00222327_m1), LAG3 (Hs00158563_m1), TBX21 (Hs00203436_m1), CXCL13 (Hs00757930_m1), and ACTB (Hs99999903_m1) (used as housekeeping control) PCR was conducted using a RotorGene Q (Qiagen) with the following conditions: initial hold for 2 min at 50 °C, enzyme activation for 10 min at 95 °C, followed by 40 cycles of 95 °C for 15 s, and 60 °C for 1 min. Data was analysed using RotorGene Q analysis software. Gene expression in ST samples relative to expression in PBMC samples was determined using the ΔΔCt method.

### Statistical analysis

For high-dimensional RNA-seq, R (version 3.5.1) and edgeR library (PMID: 19910308) was applied to analyse the sequencing data. Features were considered differentially expressed if they satisfied a twofold-change and 0.05 adjusted p value cut-off unless otherwise specified. The Benjamini–Hochberg method was used to calculate p-values adjusted for multiple hypotheses. Gene enrichment analysis was performed with Ingenuity pathway analysis (IPA) software (Qiagen, Hilden, Germany). Gene set variation analysis (GSVA) was performed in R using the “GSVA” package (PMID: 23323831). The enrichment scores were calculated using the magnitude difference between the largest positive and negative random walk deviations. Paired student t-testing were used to compare enrichment scores between groups. To analyse qRT-PCR data, GraphPad Prism 8.0 software (GraphPad, San Diego, CA) was used and gene expression tested using a two-tailed Mann–Whitney U test. Statistical significance was defined as *P* < 0.05.

### Study approval

All protocols for collecting blood and ST biopsies were approved by the Southern Adelaide Clinical Human Research Ethics Committee in accordance with the National Statement on Ethical Conduct in Human Research (2007, updated 2018; National Health and Medical Research Council Act, 1992). All patients gave written informed consent prior to participating in the study.

## Supplementary Information

Supplementary Information 1.

Supplementary Information 2.

## Data Availability

All sequencing data is publicly available in the GEO database accession number GSE199490.

## References

[1] Dennis G (2014). Synovial phenotypes in rheumatoid arthritis correlate with response to biologic therapeutics. Arthritis Res. Ther..

[2] Weyand CM, Goronzy JJ (2006). T-cell-targeted therapies in rheumatoid arthritis. Nat. Clin. Pract. Rheumatol..

[3] Fonseka CY (2018). Mixed-effects association of single cells identifies an expanded effector CD4+ T cell subset in rheumatoid arthritis. Sci. Transl. Med..

[4] Riley JL (2009). PD-1 signaling in primary T cells. Immunol. Rev..

[5] Mizuno R (2019). PD-1 primarily targets TCR signal in the inhibition of functional T cell activation. Front. Immunol..

[6] Qin W (2019). The diverse function of PD-1/PD-L pathway beyond cancer. Front. Immunol..

[7] Bartosinska J (2017). Differential expression of programmed death 1 (PD-1) on CD4+ and CD8+ T cells in rheumatoid arthritis and psoriatic arthritis. Pol. Arch. Intern. Med..

[8] Guo Y (2018). Immune checkpoint inhibitor PD-1 pathway is down-regulated in synovium at various stages of rheumatoid arthritis disease progression. PLoS ONE.

[9] Yu X (2019). BTLA/HVEM signaling: Milestones in research and role in chronic hepatitis B virus infection. Front. Immunol..

[10] Cho BA (2012). Characterization of effector memory CD8+ T cells in the synovial fluid of rheumatoid arthritis. J. Clin. Immunol..

[11] Rao DA (2017). Pathologically expanded peripheral T helper cell subset drives B cells in rheumatoid arthritis. Nature.

[12] Kostine M (2018). Rheumatic disorders associated with immune checkpoint inhibitors in patients with cancer—clinical aspects and relationship with tumour response: A single-centre prospective cohort study. Ann. Rheum. Dis..

[13] Yu M (2015). Follicular helper T cells in rheumatoid arthritis. Clin. Rheumatol..

[14] Petrelli A, van Wijk F (2016). CD8+ T cells in human autoimmune arthritis: The unusual suspects. Nat. Rev. Rheumatol..

[15] Walsh AM (2017). Triple DMARD treatment in early rheumatoid arthritis modulates synovial T cell activation and plasmablast/plasma cell differentiation pathways. PLoS ONE.

[16] Kobayashi S (2013). A distinct human CD4+ T cell subset that secretes CXCL13 in rheumatoid synovium. Arthritis Rheum..

[17] Bocharnikov AV (2019). PD-1hiCXCR5- T peripheral helper cells promote B cell responses in lupus via MAF and IL-21. JCI Insight.

[18] Andris F (2017). The transcription factor c-Maf promotes the differentiation of follicular helper T cells. Front. Immunol..

[19] Cao G (2019). CD4+CXCR5+PD-1+ T follicular helper cells play a pivotal role in the development of rheumatoid arthritis. Med. Sci. Monit..

[20] Takahashi T, Dejbakhsh-Jones S, Strober S (2006). Expression of CD161 (NKR-P1A) defines subsets of human CD4 and CD8 T cells with different functional activities. J. Immunol..

[21] Sallusto F (2000). Functional subsets of memory T cells identified by CCR7 expression. Curr. Top. Microbiol. Immunol..

[22] Linan-Rico L (2015). Analysis of expression and function of the co-stimulatory receptor SLAMF1 in immune cells from patients with systemic lupus erythematosus (SLE). Lupus.

[23] Karampetsou MP (2019). Signaling lymphocytic activation molecule family member 1 engagement inhibits T cell-B cell interaction and diminishes interleukin-6 production and plasmablast differentiation in systemic lupus erythematosus. Arthritis Rheumatol..

[24] Stephen TL (2017). SATB1 expression governs epigenetic repression of PD-1 in tumor-reactive T cells. Immunity.

[25] Beyer M (2011). Repression of the genome organizer SATB1 in regulatory T cells is required for suppressive function and inhibition of effector differentiation. Nat. Immunol..

[26] Li X, Tian F, Wang F (2013). Rheumatoid arthritis-associated microRNA-155 targets SOCS1 and upregulates TNF-alpha and IL-1beta in PBMCs. Int. J. Mol. Sci..

[27] Wu W (2019). The miR155HG/miR-185/ANXA2 loop contributes to glioblastoma growth and progression. J. Exp. Clin. Cancer Res..

[28] Nozawa K (2009). Connective tissue growth factor promotes articular damage by increased osteoclastogenesis in patients with rheumatoid arthritis. Arthritis Res. Ther..

[29] Tsai CC (2018). Essential role of connective tissue growth factor (CTGF) in transforming growth factor-beta1 (TGF-beta1)-induced myofibroblast transdifferentiation from Graves' orbital fibroblasts. Sci. Rep..

[30] Yoshitomi H, Ueno H (2020). Shared and distinct roles of T peripheral helper and T follicular helper cells in human diseases. Cell. Mol. Immunol..

[31] Kobayashi S (2016). TGF-β induces the differentiation of human CXCL13-producing CD4(+) T cells. Eur. J. Immunol..

[32] Onofrio LI (2018). Inhibitory receptor expression on T cells as a marker of disease activity and target to regulate effector cellular responses in rheumatoid arthritis. Arthritis Rheumatol..

[33] Zheng C (2017). Landscape of infiltrating T cells in liver cancer revealed by single-cell sequencing. Cell.

[34] Winkler F, Bengsch B (2020). Use of Mass Cytometry to Profile Human T Cell Exhaustion. Front. Immunol..

[35] Argyriou A (2022). Single cell sequencing identifies clonally expanded synovial CD4+ TPH cells expressing GPR56 in rheumatoid arthritis. Nat. Commun..

[36] Saeed S (2014). Epigenetic programming of monocyte-to-macrophage differentiation and trained innate immunity. Science.

[37] Petrelli A (2018). PD-1+CD8+ T cells are clonally expanding effectors in human chronic inflammation. J. Clin. Investig..

