# Supplementary material for: Transcriptomic profiling of programmed cell death 1 (PD-1) expressing T cells in early rheumatoid arthritis identifies a decreased CD4 + PD-1 + signature post-treatment
Source: Sci Rep. 2023 Feb 17;13:2847. doi: 10.1038/s41598-023-29971-5 (PMC9938264; doi:10.1038/s41598-023-29971-5)
Supplement: Supplementary file 2 — Supplementary Information 2. [file 41598_2023_29971_MOESM2_ESM.pdf]

Supplementary Material

Transcriptomic profiling of programmed cell death (PD-1) expressing T cells in early rheumatoid arthritis identifies a decreased CD4+PD-1+ signature post-treatment

Katie Lowe[1], Annabelle Small[1], Qingxuan Song[2], Ling-Yang Hao[2], William Murray-Brown[1], Susanna Proudman[3,4], Malcolm D. Smith[1], Sunil Nagpal[2], *Mihir D Wechalekar[1,5]

[1]Flinders University, Adelaide, South Australia 5042, Australia

[2]Discovery Immunology Janssen R&D, 1400 McKean Road, Spring House, PA 19477, USA

[3]Department of Rheumatology, Royal Adelaide Hospital, Adelaide, Australia

[4]Discipline of Medicine, University of Adelaide, Adelaide, Australia.

[5]Department of Rheumatology, Flinders Medical Centre, Adelaide, South Australia 5042, Australia

**Corresponding author: Mihir D. Wechalekar; mihir.wechalekar@sa.gov.au*

Number of figures: 2

Number of tables: 1

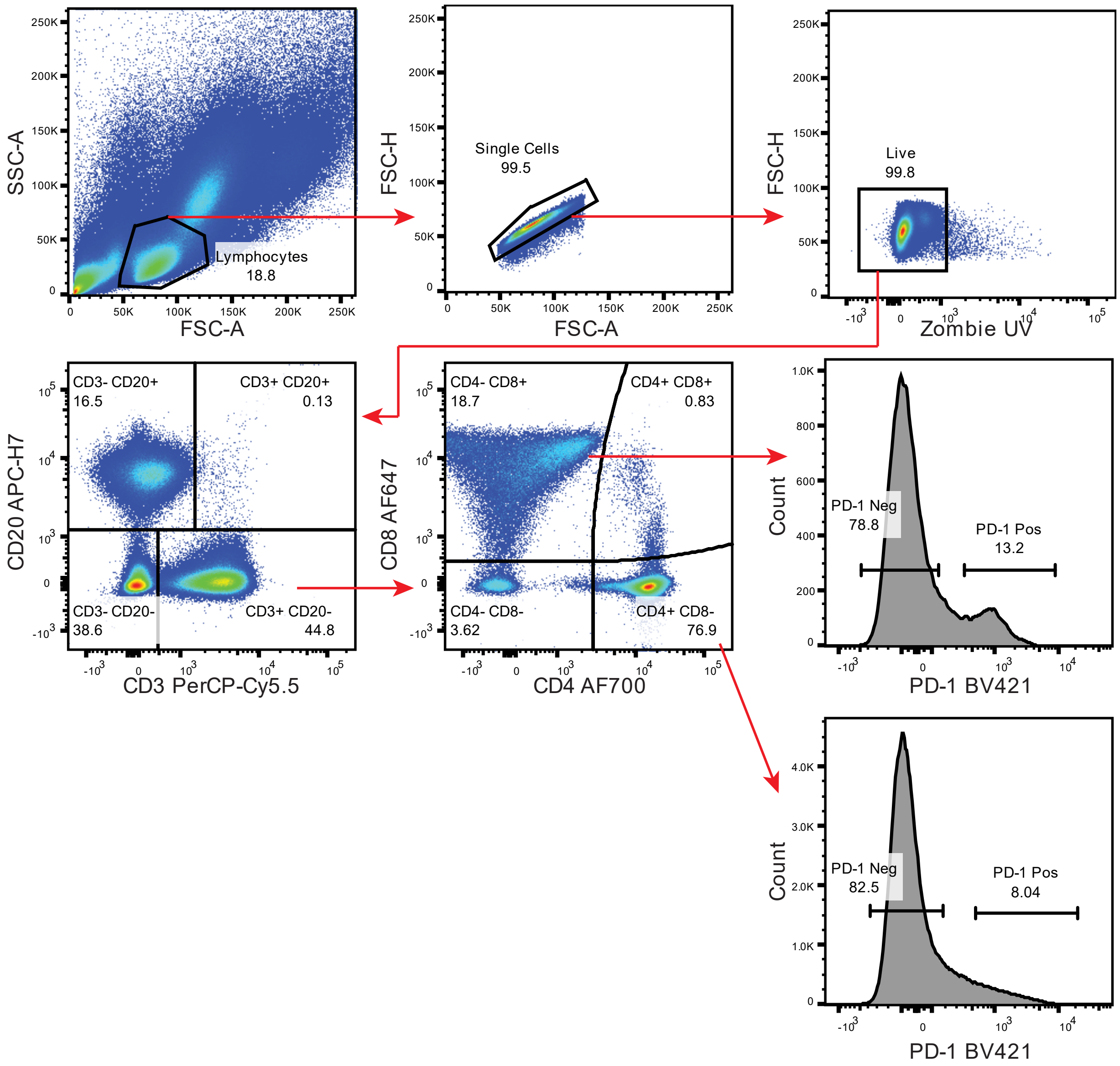

Supplementary figure 1. Representative gating strategy used for the flow cytometric sorting of CD4^{+}/CD8^{+} PD-1^{+}/PD-1^{+} populations from isolated, fresh PBMCs. Lymphocytes were gated based on size and complexity, single cells were gated and judged as viable based on their ability to exclude Zombie UV viability dye, CD3^{+} lymphocytes were gated, and from these, CD4^{+} and CD8^{+} populations were gated. Cells were then sorted based on their expression of PD-1 (positive vs. negative), resulting in the four sorted populations.

	Early RA baseline PBMC samples	Early RA baseline ST samples	Early RA 6-month ST sample
**Number of subjects**	5	19	19
**Sex—female (%)**	2 (40%)	8 (42%)	
**Age (years)**	Mean: 55<br>Range: 37-73	Mean: 50.84<br>Range: 33-72	
**Disease duration at baseline (weeks)**	Mean: 19.86<br>Range: 11-48	Mean: 17.58<br>Range: 4-40	
**CCP (U/mL)**	Min: 40 (3/5 with value >100)	Min: 11 (11/19 with value >100)	
**RF (IU/mL)**	Mean: 100.4<br>Range: 52-280<br>(1/5>900)	Mean: 130<br>Range:16-570<br>(2/19 RF negative)	
**CRP (mg/dl)**	Mean: 2<br>Range: 0.14-5.58	Mean: 2.41<br>Range: 0.069-9.2	Mean: 0.60<br>Range: 0.034-4.6
**ESR (mm/hr)**	Mean: 36.4<br>Range: 7-79	Mean: 39.42<br>Range: 7-108	Mean: 14.42<br>Range: 1-52
**DAS-28 (ESR)**	Mean: 5.19<br>Range: 4.03-6.55	Mean: 5.80<br>Range: 4.80-7.40	Mean: 3.10<br>Range: 1.08-5.56

Supplementary table 1. Patient demographics for the two cohorts used in this study. Early RA baseline PBMC samples (column 2, n=5) were sorted into $CD4^+$/$CD8^+$ PD-1^+ and PD-1^- T cell populations and subjected to bulk RNA sequencing. Early RA ST samples (n=19) baseline and post- 6-months of tDMARD treatment are shown in columns 3 and 4 (adapted from [1]).

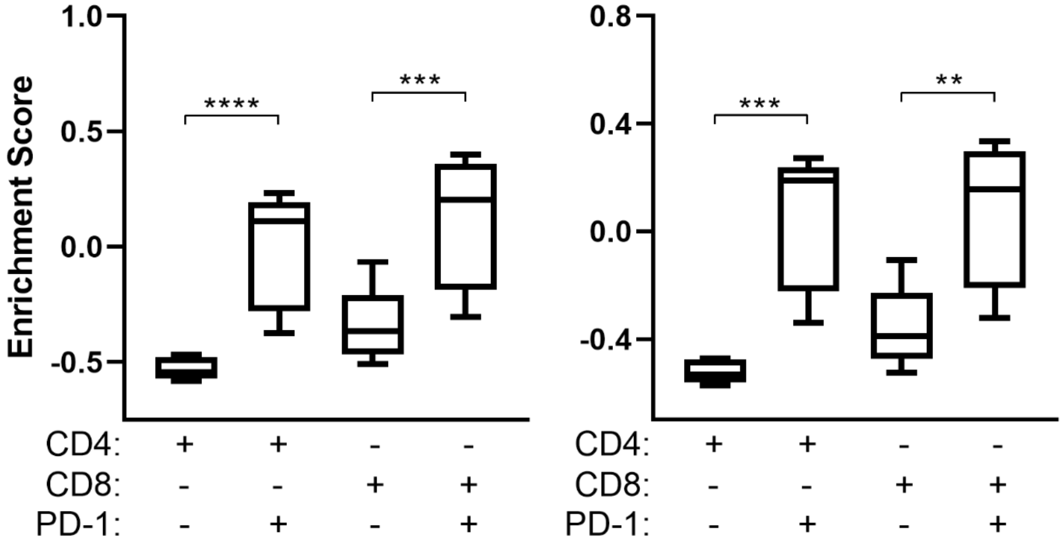

Supplementary figure 2. Exhaustion signatures are enriched in both CD4$^+$ and CD8$^+$ PD-1$^+$ cells from the peripheral blood of patients with early RA compared to PD-1$^-$ cells. Identified genes in sorted CD4$^+$/CD8$^+$ PD-1$^+$ and PD-1$^-$ cells from five patients with early RA were statistically compared with existing exhaustion gene signatures from CD4$^+$ T cells (left graph) and CD8$^+$ T cells from patients with hepatocellular carcinoma (right graph) [2]. Enrichment for exhaustion signatures are shown. Data is expressed as box and whisker plots from min to max, with box representing 25th to 75th percentile ± median. Comparisons were made by one-way ANOVA followed by Tukey's multiple comparison test, **$p<0.01$; ***$p<0.001$; ****$p<0.0001$.

References

1. Walsh, A.M., et al., *Triple DMARD treatment in early rheumatoid arthritis modulates synovial T cell activation and plasmablast/plasma cell differentiation pathways.* PloS one, 2017. **12**(9): p. e0183928-e0183928.
2. Zheng, C., et al., *Landscape of Infiltrating T Cells in Liver Cancer Revealed by Single-Cell Sequencing.* Cell, 2017. **169**(7): p. 1342-1356.e16.
